# Cytomegalovirus reactivation in autoimmune liver diseases. Can we diagnose it earlier?: A case report

**DOI:** 10.1097/MD.0000000000042876

**Published:** 2025-06-13

**Authors:** Reinaldo Cesar Silveira Filho, Sara Ribeiro Rotta, Mariana Vulcano Neres Batista, Xingshun Qi, Maria Aparecida Marchesan Rodrigues, Fernando Gomes Romeiro

**Affiliations:** a Department of Internal Medicine, São Paulo State University (UNESP), Botucatu, São Paulo State, Brazil; b Department of Gastroenterology, General Hospital of Northern Theater Command, Shenyang, China; c Department of Pathology, São Paulo State University (UNESP), Botucatu, São Paulo State, Brazil.

**Keywords:** autoimmune diseases, biliary tract diseases, cholestasis, cytomegalovirus, immunosuppression therapy, intrahepatic

## Abstract

**Rationale:**

Clinical evidence showing that biliary inflammation predisposes individuals to cytomegalovirus (CMV) disease has been growing, and some studies even indicate that this risk may surpass that associated with immunosuppression alone. Autoimmune hepatitis (AIH) can be challenging to manage and often necessitates lifelong use of immunosuppressive regimens. Additionally, AIH can be associated with chronic biliary diseases in conditions known as overlap syndromes. In one such syndrome, AIH is associated with primary sclerosing cholangitis, a well-established cause of chronic biliary inflammation that results in ductal destruction and fibrosis, thus combining 2 significant risk factors for CMV disease.

**Patient concerns:**

We report a case of a young woman with AIH and primary sclerosing cholangitis since her childhood. She was taking prednisone, azathioprine, and tacrolimus and experienced jaundice. Azathioprine and tacrolimus serum levels were adequate and AIH was well controlled, whereas total bilirubin, alkaline phosphatase, and gamma-glutamyl transferase were elevated. After the virus reactivation, the patient has been concerned about the immunosuppressive drugs she has been taken.

**Diagnoses:**

CMV reactivation was diagnosed only after hospital admission when serum polymerase chain reaction was 3153 copies/mL.

**Intervention:**

Intravenous ganciclovir resulted in rapid improvement.

**Outcomes:**

The patient achieved full recovery and no further hospitalization was needed.

**Lessons:**

Patients with similar conditions need careful vigilance for biliary CMV reactivation. Even if polymerase chain reaction is not available, CMV serology should be performed as soon as the symptoms are detected, avoiding delays in diagnosis and treatment.

## 1. Introduction

Autoimmune hepatitis (AIH) is a chronic disease that often leads to liver cirrhosis in many patients. Treatment for AIH requires the use of immunosuppressive drugs, which can cause various side effects, including an increased risk of opportunistic infections. One such infection is cytomegalovirus (CMV), also known as human herpesvirus 5. CMV can be present in up to 97% of the human population, depending on the country or region, and remains latent for life following the primary infection.^[1]^ It often causes asymptomatic or mild illness in immunocompetent adults and children, but intrauterine CMV infection can result in death in 1% of cases and lead to long-term complications in 15% to 18% of affected births.^[2]^ CMV also causes severe disease in immunocompromised patients, affecting up to 80% of solid organ transplant recipients.^[1]^

Primary sclerosing cholangitis (PSC) is a biliary disease characterized by multifocal bile duct strictures, leading to intrahepatic cholestasis and eventually resulting in liver cirrhosis. The strictures are caused by periductal fibrosis and chronic inflammation, although the underlying etiological factors remain unknown.^[3]^ Moreover, PSC is an independent risk factor for CMV reactivation after orthotopic liver transplant (OLT).^[4]^ One of the possible explanations for such an association is biliary inflammation since conditions such as biliary atresia (BA) are linked to CMV disease even with no immunosuppression.^[5]^

We present a case of a patient with overlap syndrome (AIH and PSC) who developed biliary CMV disease while undergoing immunosuppressive therapy. As the serum levels of the immunosuppressive drugs were within the normal range, PSC should lead to careful vigilance to detect biliary CMV reactivation in similar cases, performing diagnostic tests on time.

### 1.2. Ethical statements

Ethical approval was not necessary. The patient signed an informed consent giving her permission to the publication of this case report.

## 2. Case report

**Figure 1.**
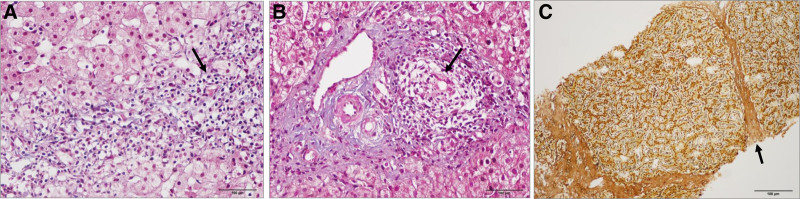
Liver biopsies showing findings of autoimmune hepatitis and primary sclerosing cholangitis. (A) Septal inflammation with spillover by lymphocytes (arrow). (B) Portal tract with lymphocytic inflammation centered on a bile duct (arrow). (C) Portal septal fibrosis (arrow). (A) Hematoxylin and Eosin stain (2004). (B) Masson trichrome stain (2007). (C) Reticulin stain (2020).

A 26-year-old woman with short stature (1.40 m) was diagnosed with AIH in 2004 at the age of 4. Liver biopsies in 2004 and 2007 were done to make the diagnosis of AIH and PSC, respectively, and revealed a lymphocytic immune attack on both liver cells and bile ducts (Fig. 1A, B), resulting in advanced liver fibrosis (F3 on the META-analysis of histological data in VIRal hepatitis score). A subsequent liver biopsy in 2020 was performed to check for AIH activity. It showed a significant reduction in bile ducts within the portal tracts, mild lymphocytic inflammation, and a similar pattern of portal–portal septal fibrosis (Fig. 1C).

A computed tomography scan in 2016 revealed focal biliary dilation in liver segment VII. During the same period, she was also diagnosed with juvenile idiopathic arthritis, which has been in remission since 2017. In 2021, magnetic resonance imaging showed multiple biliary strictures consistent with PSC, leading to a diagnosis of overlap syndrome (AIH and PSC).

The patient underwent various immunosuppressive therapies aimed at controlling AIH. In 2020, she was on a regimen of azathioprine, cyclosporine, and prednisone. This regimen was maintained until she became pregnant in 2021, at which point prednisone 30 mg alone was sufficient to maintain AIH remission. After childbirth, azathioprine was resumed, but the disease became difficult to control, requiring the addition of a third drug in January 2023. She had 45 kg and her body mass index was 22.95 kg/m^2^. At that time, azathioprine 100 mg (2.2 mg/kg/d) and prednisone 25 mg/d were combined with tacrolimus, which was introduced at 2 mg daily and increased until 6 mg/d to achieve serum levels around 6 ng/mL. Her alanine aminotransferase (ALT) levels dropped from 148 to 45 U/L (upper normal limit = 35 U/L).

However, alkaline phosphatase (ALP) and gamma-glutamyl transferase (GGT) levels increased, reaching 400 and 1304 U/L, respectively, in March 2023 (upper normal limits = 126 and 43 U/L). That same month, she developed jaundice, and her total bilirubin (TB) levels rose to 5.42 mg/dL (upper normal limit = 1.3 mg/dL). She declined hospitalization and treatment continued through outpatient appointments despite her jaundice worsening.

TB levels decreased to 2.12 mg/dL in August 2023. However, TB levels began to rise again, reaching 8.4 mg/dL in March 2024. To investigate the cause of the bilirubin fluctuations, azathioprine metabolites were measured in red blood cells. At that time, the 6-mercaptopurine level was 1128 pmol/8 × 10^8^ (reference level < 5700 pmol/8 × 10^8^), and the 6-thioguanine level was 141 pmol/8 × 10^8^ (reference range: 235–400 pmol/8 × 10^8^). Additionally, the tacrolimus level was 6.2 ng/mL. ALT, aspartate aminotransferase, and total G immunoglobulin levels were normal, indicating that AIH was well controlled and immunosuppressant levels were within safe ranges. However, ALP and GGT levels were elevated at 224 and 1190 U/L, respectively. She declined hospitalization again and jaundice worsening was maintained.

**Figure 2.**
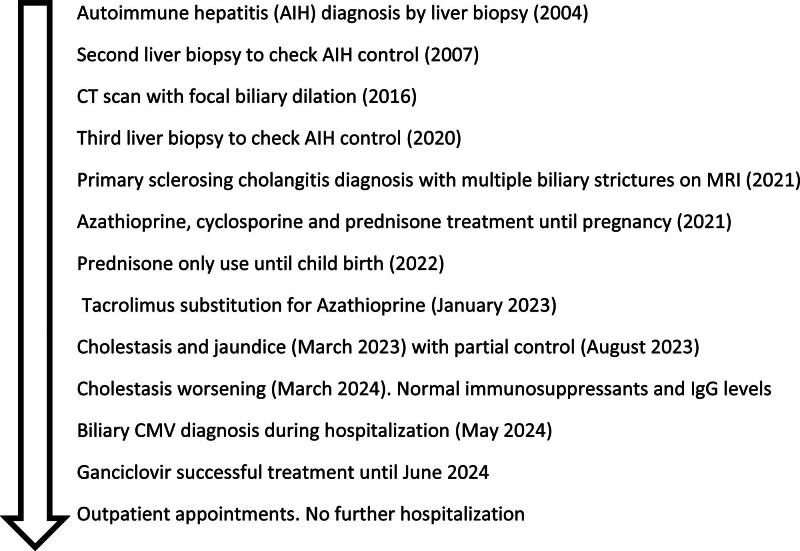
Timeline of the main events. AIH = autoimmune hepatitis, CMV = cytomegalovirus, CT = computed tomography, MRI = magnetic resonance imaging.

The patient was finally hospitalized in May 2024 due to worsening jaundice. She also presented with choluria and polyuria. Her vital signs were normal, and there were no ascites. Urinalysis revealed pyuria and hematuria. ALT levels were normal, while aspartate aminotransferase was slightly elevated. Bilirubin, ALP, GGT, urea, and creatinine levels were elevated, as detailed in Table 1. The timeline and main events are summed up in Figure 2.

**Table 1:** Serum lab tests during the hospitalization.

	D1	D3	D5	D7	D9	D11	D13
Hb (g/dL)	9.5	10.3	7.9	7.1	6.8	8.7	9.0
Plat (/mm^3^)	137,000	157,000	105,000	94,000	124,000	127,000	209,000
AST (U/L)	100	90	37	40	49	62	73
ALT (U/L)	138	135	64	47	44	54	72
GGT (U/L)	780	653	438	438	444	556	665
ALP (U/L)	255	-	-	-	224	249	232
TB (mg/dL)	12.9	16.6	15.2	14.1	10.9	8.8	9.2
DB (mg/dL)	6.7	9.7	8.9	8.5	5.6	3.9	3.9
Cr (mg/dL)	2.4	2.6	2.7	3.1	2.4	2.4	2.2
Ur (mg/dL)	99	129	127	105	86	76	78

ALP = alkaline phosphatase, ALT = alanine aminotransferase, AST = aspartate aminotransferase, Cr = creatinine, D1–D13 = hospitalization days 1 to 13, DB = direct bilirubin, GGT = gamma-glutamyl transferase, Hb = hemoglobin, Plat = platelets count, TB = total bilirubin, Ur = urea.

Abdominal ultrasonography showed no signs of portal hypertension, and her kidneys and biliary tract did not exhibit additional disease-related abnormalities. Serological tests for opportunistic infections revealed a CMV polymerase chain reaction (PCR) level of 3153 copies/mL (3.5 log).

She was treated with intravenous ganciclovir (100 mg daily for 3 weeks), resulting in rapid improvement. A follow-up CMV PCR test performed 1 week after starting ganciclovir showed no detectable virus.

Ganciclovir treatment continued for 3 weeks, but the hospitalization lasted only 13 days as the patient chose to leave the hospital and receive the remaining treatment in an outpatient care facility. She has recovered from the infection and did not need further hospitalization.

## 3. Discussion

Patients with AIH often require lifelong immunosuppressive therapy, which can lead to opportunistic infections. PSC and overlap syndromes are rare and the incidence of CMV infection in these conditions is not well known, making it challenging to assess risk factors. However, data from other immunosuppressive conditions and biliary diseases are more consistent and available.

A large study on rheumatic diseases found that 2% of hospitalized patients experienced CMV reactivation. Nearly all of these patients were on corticosteroids, with many also receiving other immunosuppressive agents. Reactivation occurred in patients undergoing strong immunosuppressive therapies, and corticosteroid use not only increased the risk of CMV reactivation but also the mortality risk during the infection.^[6]^ A similar association has been observed in patients receiving solid organ transplants, for whom the degree of immunosuppression is also a risk factor.^[1]^ Our patient was on 3 immunosuppressive drugs, which were within safe levels. Although the exact timing of her viral reactivation is unclear (because CMV PCR is only available in hospitals), it likely began when the jaundice appeared. This assumption is based on the fact that she had never experienced jaundice before and that the jaundice resolved quickly after starting ganciclovir. Moreover, it did not relapse after CMV treatment. In similar cases, we recommend testing for CMV-IgM antibodies as soon as jaundice develops, as this is a cost-effective test that can be performed in outpatient settings.

Nevertheless, immunosuppression is not the only risk factor for CMV disease. A recent study found that one-third of children with BA who underwent Kasai portoenterostomy developed signs of CMV infection.^[5]^ The authors make intriguing insights into the potential role of CMV in the pathophysiology of BA. They note that 9.5% to 38% of children with BA in various Western countries had serum CMV-IgM antibodies, with the prevalence rising to 60% in China, where the incidence of BA is higher.^[7,8]^ The association between the diseases was further evaluated in a meta-analysis, which found that patients with BA who also have CMV experience a higher degree of inflammation, more severe liver fibrosis, and worse outcomes.^[8]^ Unlike the patients in these studies, the patient described here is an adult with mature immunological development, a dissimilar timing of CMV infection, and a different biliary disease. However, both BA and PSC cause biliary inflammation and fibrosis, making the biliary tract susceptible to CMV disease.

Finally, a German study of 833 patients who underwent OLT demonstrated that PSC is an independent risk factor for CMV infection after surgery. This finding indicates that PSC increases susceptibility to CMV under immunosuppressive conditions. The study reported an odds ratio of 3.76 for CMV infection in patients with PSC, suggesting that PSC may be a more significant risk factor than the well-known association of donor-positive and recipient-negative serology for CMV. The authors explained that the study differentiated between CMV infection, which may be asymptomatic, and CMV disease, which presents with symptoms. All patients included were receiving tacrolimus either as monotherapy or in combination with mycophenolate mofetil, and PSC was present in only 8% of the cohort.^[4]^

It is important to note that our patient developed jaundice only after starting tacrolimus, which intensified her immunosuppression. Tacrolimus is not an independent risk factor for CMV disease after OLT. However, a 3-drug regimen (odds ratio = 6.7 compared to a 2-drug regimen) and cyclosporine (odds ratio = 6.7 compared to tacrolimus) are independent risk factors for CMV reactivation after OLT.^[9]^ Thus, the destruction of biliary ducts observed in all liver biopsies of our patient suggests that this finding, combined with the 3-drug immunosuppression, was a key factor in her CMV reactivation.

One of the main strengths of this report is the thorough documentation of liver biopsies from the patient’s childhood, which illustrates that her liver fibrosis worsened faster before the AIH diagnosis but was effectively controlled afterward, preventing the development of portal hypertension. Another advantage is that azathioprine and tacrolimus serum levels were registered at the time of the viral reactivation. However, a limitation of the report is the absence of CMV testing at the onset of jaundice, which prevents precise determination of when the reactivation occurred. Additionally, changes in immunosuppressive therapy, which are common in AIH treatment, also occurred. Despite this, the clinical data described support a timely correlation between viral reactivation and the onset of jaundice.

In conclusion, patients with AIH and PSC need a careful vigilance for CMV reactivation due to their biliary changes and their need of immunosuppression. We encourage the publication of similar case reports and suggest that CMV serology be performed earlier in cases of jaundice to facilitate the prompt detection of viral reactivation.

## Acknowledgments

The authors would like to acknowledge Botucatu Medical School and Sao Paulo State University (UNESP).

## Author contributions

**Data curation:** Reinaldo Cesar Silveira Filho, Sara Ribeiro Rotta, Mariana Vulcano Neres Batista, Maria Aparecida Marchesan Rodrigues, Fernando Gomes Romeiro.

**Formal analysis:** Reinaldo Cesar Silveira Filho, Sara Ribeiro Rotta, Maria Aparecida Marchesan Rodrigues, Fernando Gomes Romeiro.

**Investigation:** Reinaldo Cesar Silveira Filho, Sara Ribeiro Rotta, Mariana Vulcano Neres Batista, Maria Aparecida Marchesan Rodrigues, Fernando Gomes Romeiro.

**Writing—original draft:** Reinaldo Cesar Silveira Filho, Xingshun Qi, Fernando Gomes Romeiro.

**Project administration:** Xingshun Qi, Fernando Gomes Romeiro.

**Supervision:** Xingshun Qi, Maria Aparecida Marchesan Rodrigues, Fernando Gomes Romeiro.

**Writing—review & editing:** Xingshun Qi, Maria Aparecida Marchesan Rodrigues, Fernando Gomes Romeiro.

**Conceptualization:** Maria Aparecida Marchesan Rodrigues, Fernando Gomes Romeiro.
